# Psychotic symptoms in Chinese patients with somatic symptom disorder: prevalence, risk factors, and associated conditions

**DOI:** 10.3389/fpsyt.2025.1519492

**Published:** 2025-02-26

**Authors:** Jiamei Yuan, Yunhui Zhong, Yibo Li, Yuanping Liao, Hong Tang

**Affiliations:** ^1^ College of Humanities and Social Sciences, Gannan Medical University, Ganzhou, China; ^2^ Department of Psychology, The Third People’s Hospital of Ganzhou, Ganzhou, China; ^3^ Department of Psychiatry and Psychology, College of Basic Medical Sciences, Tianjin Medical University, Tianjin, China; ^4^ Department of Geriatric, The Third People’s Hospital of Ganzhou, Ganzhou, China

**Keywords:** psychotic symptoms, somatic symptom disorder, risk factors, anxiety, depression, stress

## Abstract

Psychotic symptoms are prevalent in individuals with various mental health disorders and frequently lead to adverse outcomes. In this study, we assessed the prevalence of psychotic symptoms and its associated conditions in a large sample of Chinese patients with somatic symptom disorder (SSD), which has not been examined systemically. We recruited 899 patients with SSD. We used the positive subscale of the Positive and Negative Syndrome Scale to assess psychotic symptoms in the participants. We evaluated the participants using the Hamilton Depression Rating Scale (HAMD), Hamilton Anxiety Rating Scale (HAMA) and Perceived Stress Scale (PSS).The prevalence of psychotic symptoms in participants was 10.2%. Compared with participants without psychotic symptoms, participants with psychotic symptoms had higher scores on the HAMD, HAMA and PSS scales and a shorter sleep duration. Based on the results of stepwise binary logistic regression analysis, the HAMA, HAMD and PSS were significantly associated with psychotic symptoms in the participants. Our findings suggest that psychotic symptoms are common in patients with SSD in the Chinese Han population. In addition, greater levels of anxiety, depression, and stress are potentially useful markers for predicting a greater risk of psychotic symptoms.

## 1 Introduction

According to the Diagnostic and Statistical Manual of Mental Disorders, Fifth Edition (DSM-5), somatic symptom disorder (SSD) is the prototypical diagnosis for patients with somatic symptom and related disorders (1). Its major characteristics include somatic distress, catastrophizing cognitive behavior, health-related anxiety, and exaggerated reactions to somatic discomforts (2). SSD is a mental health condition reported in clinical settings, both in China and in other countries. This condition is widely reported in primary care in Western countries, with prevalence estimates ranging from 11.7% to 30.3% in studies conducted between 1999 and 2022 (3–6) In a 2020 survey of outpatients in China, the prevalence of DSM-IV SSD was found to be 33.8% (236/697) (7). This indicates how significant the issue is globally.

Even though the symptoms of SSD cannot be directly attributed to an organic pathology, patients with SSD are at a higher level of disability risk than patients with other conditions that are known to have greater disability risk than SSD (8). This not only restricts the social engagement of patients (9) but also profoundly affects their functional capabilities and overall quality of life (10). This causes physical and mental exhaustion in patients. Furthermore, because patients tend to use healthcare resources more frequently (8, 11), they incur significant health expenses, which increases the economic burden as well (9, 12). Thus, this condition also exerts considerable pressure on medical systems (13, 14). Reports indicate that, in the United States, the healthcare expenses for patients with SSD are six to fourteen times greater than the average healthcare expenses (15). Consequently, the effective clinical management of patients with SSD is a critical challenge in the mental healthcare system and requires urgent attention, given its inherent complexity and significance (16, 17).

Several studies have shown that the prevalence of a mental health condition along with other psychotic symptoms often correlates with more severe manifestations, elevated levels of suicidal ideation (18), anxiety (19), hypochondriasis (20), cognitive dysfunction (21), and A-type personality disorder (22), as well as poorer prognoses and higher rates of treatment resistance, among other adverse outcomes. Patients with SSD exhibit personality traits that predispose them to psychotic symptoms (23), characterized by heightened neuroticism (24) and signs of punishment sensitivity (25). Consequently, when patients with SSD display additional psychotic symptoms, they are likely to experience more severe repercussions than patients who have an isolated episode of SSD.

The increase in comorbidities of psychotic symptoms in SSD is associated with multifaceted mechanisms. Substantial evidence indicates that anxiety and depression may serve as potential etiological factors (26) and are common complications in patients with SSD. These negative emotional states heighten the risk of psychotic symptoms, frequently exacerbating both the severity and distress associated with such symptoms (27). Furthermore, SSD and psychotic symptoms have similar pathogenic mechanisms, with heightened life stress being a common causative factor (28, 29). The origins of this association remain unclear, but there may be potentially overlapping genetic risk factors that influence stress perception in patients with psychiatric disorders, with stress sensitivity being heritable (30). In previous studies, variables such as sex (31) and age (32, 33) have also been shown to influence the manifestation of psychotic symptoms, and therefore, they are potential risk factors that we need to consider.

Patients with both somatic symptom disorders and psychotic symptoms may experience more severe manifestations and additional adverse outcomes. However, the prevalence and risk factors associated with comorbid psychotic symptoms in patients with SSD has not been investigated sufficiently. Here, we examine the prevalence of psychotic symptoms in patients with SSD and the risk factors contributing to this prevalence.

## 2 Methods

### 2.1 Study setting and participants

We conducted a cross-sectional survey from January 2023 to April 2024 in The Third People’s Hospital of Ganzhou, Ganzhou People’s Hospital, and First Affiliated Hospital of Gannan Medical University, Jiangxi Province, China. The study protocol was approved by the Institutional Review Board of The Third People’s Hospital of Ganzhou. We obtained informed consent from all patients before requesting their participation in this study. The information provided by all respondents was confidential.

We recruited 899 participants from the outpatient departments of the psychiatry wards at The Third People’s Hospital of Ganzhou, Ganzhou People’s Hospital, and the First Affiliated Hospital of Gannan Medical University. The inclusion criteria were as follows: (1) Chinese Han nationality, based on self-reporting by the participants; (2) age of participants: 18-60 years; (3) diagnosis of SSD according to the Diagnostic and Statistical Manual of Mental Disorders (DSM-5) criteria; (4) ability of participants to provide written informed consent. Before we assigned definitive diagnoses, we discussed them in our weekly team meeting, and a supervising senior physician with extensive clinical experience validated the diagnoses. Eight hundred and ninety-nine patients met the inclusion criteria. Ninety-eight patients were excluded for the following reasons: Ninety-eight patients were excluded for the following reasons: (1) they were pregnant or lactating (n=23); (2) they had substance use disorder (n=25), which was diagnosed according to DSM-5 criteria for substance use disorder; (3) they had severe personality disorder (n=13), as diagnosed using the DSM-5 criteria for severe personality disorders;(4) they had severe physical diseases (n=12); (5) they refused to participate in the study (n=20); (6) they were excluded for other unknown reasons (n=5).

### 2.2 Demographic data collection

Trained researchers systematically distributed questionnaires to each participant to gather comprehensive information, including their age, gender, educational background, marital status, body mass index (BMI), age of disease onset, and duration of disease. We meticulously reviewed the available medical records of participants. To address any missing data or ambiguous responses, we conducted supplementary interviews with relatives or attending physicians.

### 2.3 Clinical measurement

We used the positive subscale of the Positive and Negative Syndrome Scale (PANSS) to identify psychotic symptoms (34). It comprises seven items scored on a 7-point scale. The total score ranges from 7 (absent) to 49 (high) (extremely severe). Participants were considered to have psychotic symptoms if their score was greater than or equal to 15. Participants with a score less than or equal to 14 were considered to have no psychotic symptoms (34).

We used the 17-item Hamilton Depression Rating Scale (HAMD) (35) to evaluate the level of depression in patients. This scale comprises 17 items, including eight items rated on a five-point scale (0: not present, 4: severe) and nine items rated on a three-point scale (0: not present, 2: severe). We determined the presence and severity of depression based on the cumulative HAMD score. The Chinese version of this scale has been validated for its reliability and validity (35).

We used the 14-item Hamilton Anxiety Rating Scale (HAMA) (36) to assess the anxiety levels of participants. This scale comprises 14 items, measured using a five-point Likert scale (0: not present, 4: severe), with a maximum score of 56 points. We assessed the presence and severity of anxiety based on the total HAMA score. The Chinese version of this scale also has good reliability and validity (36).

The Perceived Stress Scale (PSS) (37) is used to measure stress levels in participants. The PSS has seven items, all of which are rated on a five-point Likert scale (0: not present, 4: severe). We instructed the participants to record their responses promptly based on their honest opinions about a particular event in the preceding month. We assessed the existence and degree of stress based on the total PSS score.

Two experienced psychiatrists with specialized training collected the above information. They had no prior knowledge of the clinical data of the participants. After repeated evaluation, the observer correlation coefficients of the HAMD total score and HAMA total score were found to be greater than 0.8.

### 2.4 Data analysis

For data analysis, we used the Kolmogorov Smirnov single-sample test to perform normality test on the data of each variable. The data were not normally distributed. When we compared the demographic and clinical variables between groups with and without symptoms of mental illness, we used the Mann-Whitney U test for continuous variables and the Chi-square test for categorical variables. We performed binary logistic regression analysis using factors closely related to psychotic symptoms, with gender and age included as covariates. We conducted statistical analyses using SPSS version 23.0.

## 3 Results

### 3.1 Demographic characteristics of patients with somatic symptoms disorder

Our study had 899 patients (579 females, 320 males). The median age of the patients was 33 years (range: 18 to 60 years). Of the patients, 597 had a bachelor’s degree or less advanced degree, and 302 patients had a bachelor’s degree or more advanced degree. The median age of illness onset was 32 years (range: 17 to 60 years). The median duration of illness was 11 years (range: 6.5 to 32 years). There were no significant differences in these demographic characteristics between the two groups of patients with and without psychotic symptoms.

### 3.2 Prevalence of psychotic symptoms in patients with somatic symptoms disorder

**Figure 1:**
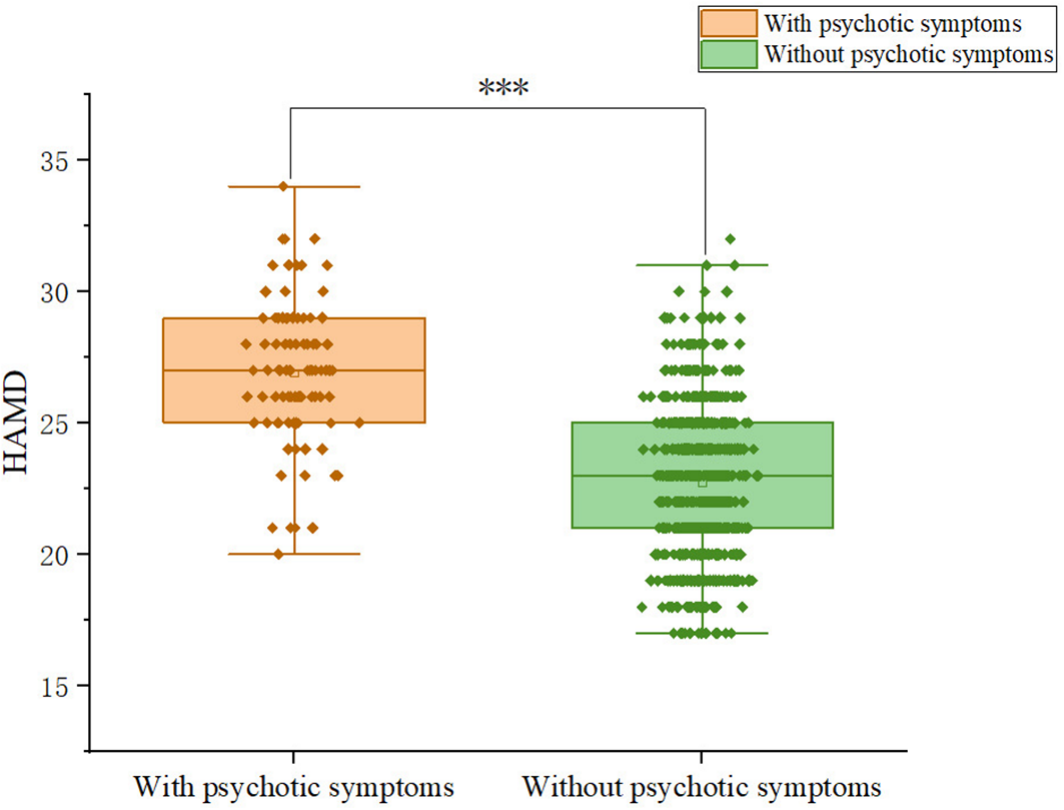
Differences in HAMD scores between participants with and without comorbid psychiatric symptoms.

**Figure 2:**
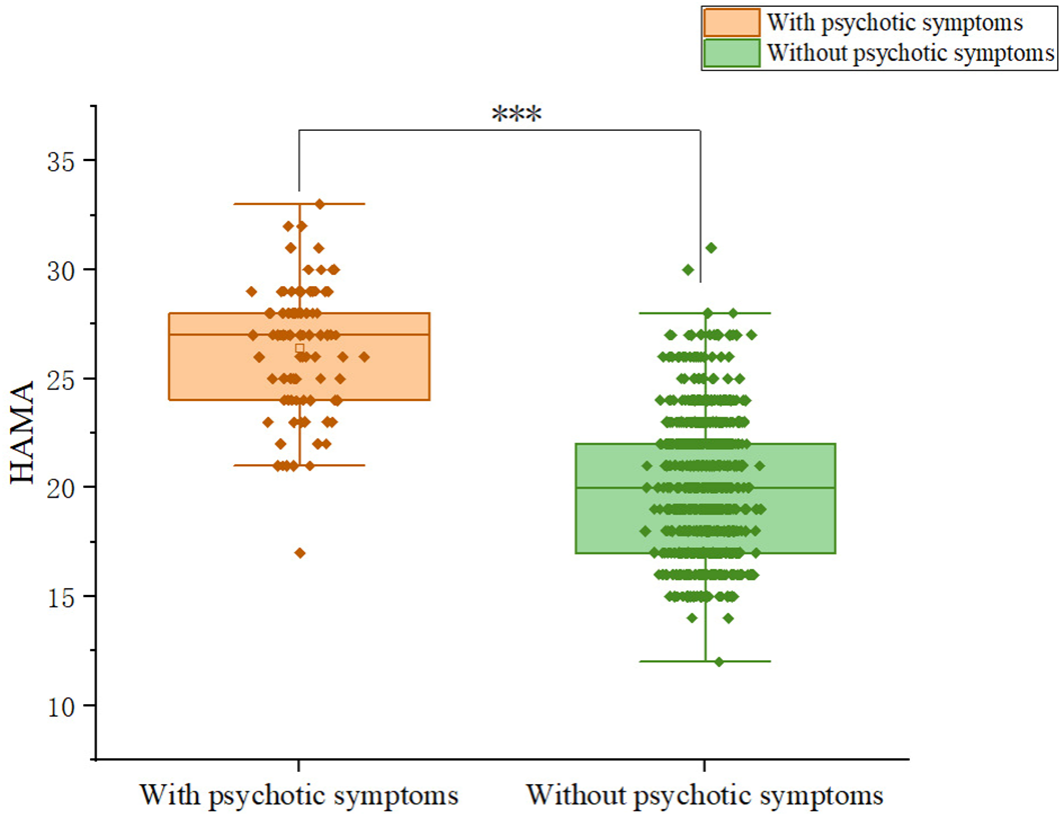
Differences in HAMA scores between participants with and without comorbid psychiatric symptoms.

**Figure 3:**
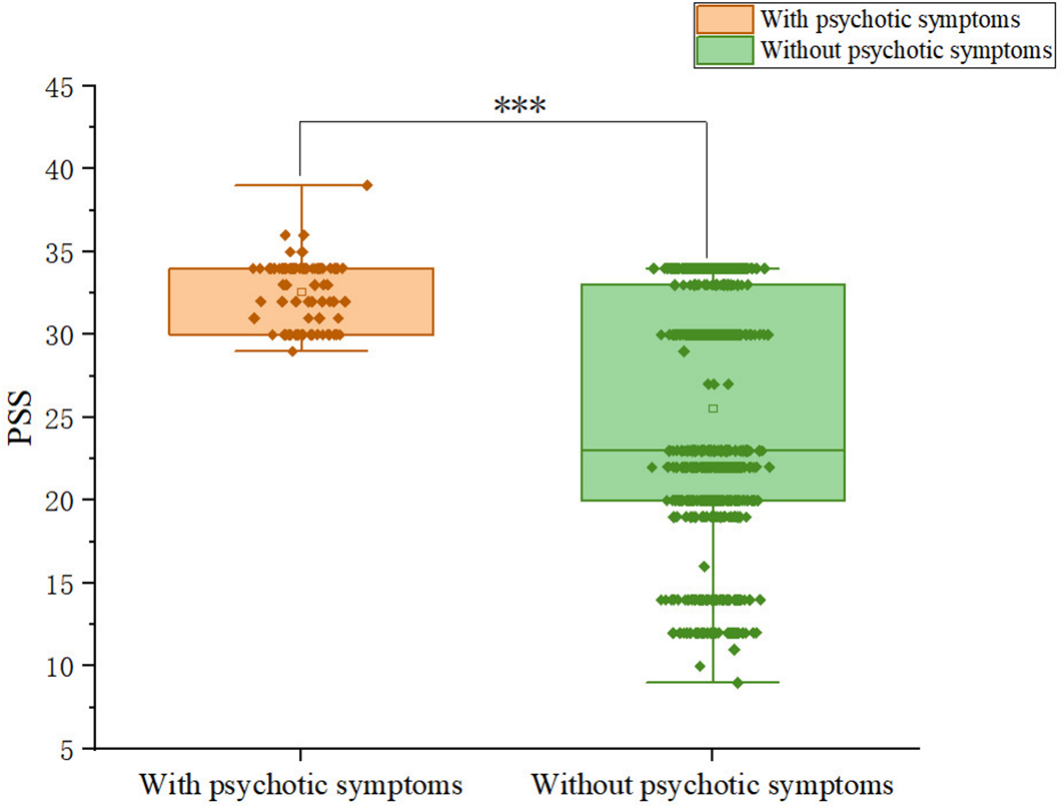
Differences in PSS scores between participants with and without comorbid psychiatric symptoms.

Patients with somatic symptoms disorder had a 10.2% (92/899) prevalence of psychotic symptoms. Among patients, 9.59% (28/292) of males and 12.42% (64/515) of females exhibit psychotic symptoms; the difference was not significant (χ^2^ = 1.19, p = 0.275).We compared and presented the demographic and clinical characteristics of patients with and without psychotic symptoms in **Table 1**. We observed significant differences between the psychotic and non-psychotic patient groups with respect to the following variables: HAMD (Z = -11.374, p<0.001), HAMA (Z= -13.577, p<0.001), PSS total score (Z=-9.394, p<0.001). Patients with psychotic symptoms exhibited higher levels of anxiety and depression than patients without psychotic symptoms. In addition, patients with psychotic symptoms experienced more severe stress than patients without psychotic symptoms. **Figures 1**-**3** display the significant differences in HAMD, HAMA, and PSS total scores, with the corresponding levels of significance marked.

**Table 1:** Characteristic of somatic symptom disorder patients with or without psychotic symptoms.

	Psychotic Median (IQR) n=92	Non-psychotic Median (IQR) n=807	Z or X^2^	p
Age (years)	33 (26)	32 (21)	-0.665	0.506
**Education level**			0.828	0.363
Below undergraduate	65	532		
Bachelor or above	27	275		
Gender (f/m)	64/28	515/292	1.19	0.275
**Marital status**			0.082	0.774
Unmarried	31	260		
Married	61	547		
Age of onset (year)	33 (26)	32 (20)	-0.693	0.488
Duration of illness (years)	12 (4)	11 (5)	-1.549	0.121
BMI	24.325 (2.84)	24.18 (2.41)	-1.342	0.180
HAMD	27 (4)	23 (4)	-11.374	<0.001
HAMA	27 (4)	20 (5)	-13.577	<0.001
PSS	34 (4)	23 (13)	-9.394	<0.001

“Education level” and “Marital status” are categorical variables, bolded to distinguish them from their categories.

### 3.3 Risk factors for psychotic symptoms in patients with somatic symptoms disorder

We performed a stepwise forward binary logistic regression to identify the risk factors for psychotic symptoms. After controlling for the covariates of gender and age, the following variables reached statistical significance: The HAMA score (odds ratio = 1.175, 95% CI = 1.022—1.352, Wald χ^2^ = 71.927, p<0.001), HAMD score (odds ratio = 1.175, 95% CI = 1.022—1.352, Wald χ^2^ = 5.11 p = 0.024), PSS total score (odds ratio = 1.170, 95% CI = 1.044—1.312 Wald χ^2^ = 7.268, p = 0.007), were important predictors for psychotic symptoms in patients. The VIF values of the independent variables in the model are all less than 5, indicating that there is no significant multicollinearity issue among the predictors (35). **Table 2** showed the results of the logistic regression.

**Table 2:** Binary logistic regression in somatic symptom disorder patients with psychotic symptoms.

	Coefficient B	Std.error	Wald	P value	Exp (B)	lower	upper	VIF
HAMD	0.162	0.071	5.11	0.024	1.175	1.022	1.352	2.311
HAMA	0.548	0.065	71.927	<0.001	1.730	1.524	1.963	1.669
PSS	0.157	0.058	7.268	0.007	1.170	1.044	1.312	2.029

## 4 Discussion

To the best of our knowledge, this is the first study to identify the prevalence and risk factors of psychotic symptoms in Chinese Han patients with somatic symptoms disorder. We observed concomitant psychotic symptoms in 10.2% of patients with SSD. Additionally, some clinical characteristics, such as greater HAMD,HAMA and PSS scores were identified as risk factors for psychotic symptoms.

The prevalence rate of psychotic symptoms was higher in our sample than in some studies reporting the combined rate of other psychiatric disorders and psychotic symptoms (38). Compared to the prevalence of other comorbidities in patients with SSD, the prevalence of psychotic symptoms in patients with SSD surpasses the combined prevalence of SSD with depression (4.1%) and anxiety (5.5%) (39). This indicates that patients with SSD patients experience greater complications when they also experience psychotic symptoms; this warrants greater attention from researchers. To investigate the high co-incidence of psychotic symptoms in patients with SSD, we combined data from previous studies and arrived at the following plausible explanations. First, patients with SSD are more likely to be hypersensitive and react more severely to physical symptoms than patients with other mental health conditions (40). Some findings indicate that hallucinations are a response to hypervigilance (41, 42). Patients with SSD are prone to mental health symptoms, probably owing to their hypervigilance toward physical symptoms. Second, patients with SSD repeatedly seek medical treatment without being able to identify the cause (43) and repeatedly experience setbacks. Patients with SSD also exhibit a weak coping ability (44) and resilience (45). Also, because they do not have substantial coping ability and mental resilience to deal with such setbacks, they are more prone to psychotic symptoms (46).

Anxiety and depression are significantly correlated with psychotic symptoms (27, 47, 48)and represent the most prevalent complications among patients with SSD (49–51). Our research further elucidated that anxiety and depression are predictors of psychotic symptoms in patients with SSD. The predictive effects of anxiety and depression on psychotic symptoms have been extensively validated in previous studies (52–54). For instance, Machado et al. conducted a three-year longitudinal study demonstrating that symptoms of childhood anxiety in populations at a high risk for psychosis can serve as predictors of psychotic symptoms during adolescence (55). Smith et al. discovered that a greater severity of depression correlates with the intensity of auditory hallucinations and delusions of victimization (56). The mechanisms by which depression and anxiety influence psychotic symptoms have been reported in some studies. Morrison et al. (57) posited from a cognitive standpoint that anxiety and depression play a direct role in both the development and persistence of delusions and hallucinations. They stated that negative emotions may cause patients to misinterpret fundamentally regular experiences as threatening events, making them feel distressed and experience psychotic phenomena. This fosters a detrimental cycle of adverse emotions, physiological alterations, and safety-seeking behaviors in affected individuals. Bental et al. (58) reported an alternative explanation based on psychological defense mechanisms. They proposed that delusions—particularly those characterized by persecutory themes—are a defensive response to low self-esteem and depression. In summary, anxiety and depression not only represent prevalent mental health challenges for patients with SSD but also serve as significant predictors for psychotic symptoms. This finding highlights the importance of focusing on and effectively managing the emotional well-being of such patients during therapeutic interventions for SSD. Treatment strategies should include comprehensive psychological evaluations aimed at accurately identifying and assessing levels of anxiety and depression in such patients.

The experience of stress is frequently closely linked to the development of various mental health conditions (59–61). The findings from this study substantiate this notion and indicate that stress is a pivotal factor in forecasting psychotic symptoms in patients with SSD. Previous research findings have elucidated the underlying mechanisms through which stress influences psychotic symptoms across multiple dimensions, including psychological, physiological, and genetic factors. The stress-vulnerability model proposed by Zubin and Spring (62) underscores that experiences of stress play a crucial role in precipitating acute psychotic episodes. Specifically, inadequately managed stressful events, coupled with significant distress and anxiety, may exacerbate psychotic symptoms in individuals who are highly susceptible. Furthermore, stress can impact mental health via biological pathways. Psychosocial stressors can disrupt the functional equilibrium of the hypothalamic-pituitary-adrenal axis (63) and potentially influence neurotransmitter transmission (64). These biological alterations are integral to understanding how stress contributes to the pathological processes associated with mental disorders (65). From a genetic standpoint, evidence suggests the existence of familial predisposition of sensitivity to stress responses as a risk factor for psychotic symptoms (66). This sensitivity may be inherited through genetic mechanisms (30, 67). Given the multifaceted impact of stress on psychiatric manifestations spanning various domains, strengthening interdisciplinary collaboration among psychiatry, neurology, psychology, and other relevant fields to collectively deliver comprehensive medical services to affected patients is recommended.

In this study, we systematically investigated the prevalence of psychotic symptoms in patients with SSD, drawing on a large clinical data sample from China, and explored the potential predictive factors. This area of research has received relatively limited attention in research but holds significance in clinical practice. Our findings aim to enrich the investigations in this field by providing detailed and representative data. Additionally, these findings are closely linked to clinical practice and may offer more precise guidance for doctors in their daily work, aiding the identification and assessment of and interventions for psychotic symptoms in patients with SSD. This would help positively influence the rehabilitation process.

We observed several limitations of this study. First, the absence of healthy controls matched for age and gender with the participants is a significant limitation, which may have introduced bias when comparing our patient cohort with those from other studies. Second, due to the cross-sectional nature of our study, we were unable to establish causal relationships between clinical variables and psychotic symptoms in patients with somatic symptom disorder (SSD). Third, various genetic and environmental factors, such as genetic predisposition, levels of brain-derived neurotrophic factors, physical activity, and a family history of psychotic symptoms, may influence these symptoms. However, we did not collect such data in our investigation. Additionally, the sampling scope of this study was limited to Jiangxi Province, and future studies should aim to collect more representative data from a wider range of regions to enhance the generalizability of the findings. Furthermore, this study did not address the potential impact of socioeconomic status and healthcare accessibility on psychotic symptoms, which is an important factor for future investigations. In future research, large-scale controlled prospective studies should be conducted to better elucidate the relationship between these factors and psychotic symptoms.

In summary, the findings of this study revealed a 10.2% prevalence of psychotic symptoms in patients with SSD, suggesting that psychotic symptoms are common in patients with SSD in the Chinese Han population. Furthermore, our findings showed that greater levels of anxiety, depression and stress (indicated by high HAMA, HAMD, PSS) are risk factors for psychotic symptoms in patients with SSD. Psychotic symptoms result in increased anxiety, depression, and stress levels. Understanding the risk factors of psychotic symptoms can help identify their implications. This can help develop interventions and preventive methods and reduce the burden of psychotic symptoms in patients with SSD.

## Data Availability

The original contributions presented in the study are included in the article/supplementary material. Further inquiries can be directed to the corresponding author.
